# Pilot educational program to enhance empowering patient education of school-age children with diabetes

**DOI:** 10.1186/2251-6581-12-16

**Published:** 2013-05-06

**Authors:** Marjatta Kelo, Elina Eriksson, Ilse Eriksson

**Affiliations:** 1 Institute of Behavioural Sciences, University of Helsinki, Siltavuorenpenger 5 A, P.O. Box 9, Helsinki, 00140, Finland; 2 Department of Nursing Science, University of Turku, Turku, Finland

**Keywords:** Patient education, Empowerment, Children, Diabetes

## Abstract

**Background:**

Nurses have a crucial role in patient education of children with type 1 diabetes, but they often exhibit lack of knowledge of the patient education process. This study aimed to describe an educational program to enhance empowering patient education process for the blood glucose monitoring education of school-age children and nurses’ perceptions of using empowering techniques.

**Methods:**

An empowering patient education process for the diabetes education of school-age children was developed. The researcher collected nurse’s perceptions of managing the educational program by semi-structured interviews. Ten nurses carried out the diabetes education, and 8 of them participated in the interview. Three nurses implemented the diabetes education twice and were interviewed twice. The data consisted of 11 descriptions of the blood glucose monitoring education. The interviewer analyzed the data deductively and inductively by content analysis.

**Results:**

Nurses described successful managing of the empowering patient education process. The need assessment consisted of using multiple methods and clarifying the capabilities and challenges of children and their parents. Planning manifested itself in adequate preparation and multiple objectives stated together with the family. Implementation comprised the relevant content, and the use of suitable teaching materials and methods. Evaluation was performed with various methods and documented accurately. Nurses also faced some challenges related to management and leadership, ambivalence with traditional and empowering patient education, and families’ overall situation.

**Conclusion:**

An example of developing evidence-based patient education program is presented, but besides education other factors supporting changes in work practices should be considered in further development.

## 

Type 1, insulin-dependent diabetes is one of the most common chronic diseases in childhood. Diabetes management relies on self-management [1,2]. Therefore, patient education is a key intervention in promoting family health when a child is diagnosed with type 1 diabetes. The goal of diabetes education is to empower children and their parents. In other words, they acquire knowledge and skills as well as increase self-awareness to master and control the treatment and their lives [3,4]. At school-age, children can learn diabetes self-management skills, but they need their parents to share the responsibility for the diabetes management [5].

The education and treatment of children with diabetes is carried out by a multidisciplinary team [2,6], but nurses have a crucial role in diabetes education [7,8]. However, they often exhibit lack of knowledge of the patient education process [9]: teaching is not always based on the assessment of individual needs [10], education is conducted without setting clear goals for teaching [11], and it is nurse-oriented [12]. Moreover, the use of evaluation strategies and documentation is limited [10,11].

A patient education process includes assessment, planning, implementation, and evaluation [9,13]. In this process, empowering education emphasizes the whole family and its strengths, children’s and parents’ learning needs, shared goals, individualized knowledge, family-driven decision-making and promotion of participation [4,14]. If these elements are not involved, patient education is provided traditionally, in a nurse-oriented way. Empowering patient education accentuates the cooperation with the child and parents in each phase of the process. Empowering patient education has already been part of nursing for a long time, but when nurses’ experiences of patient education were studied, both empowering and traditional education emerged [12]. In addition, school-age children’s perceptions of patient education revealed that their participation in it was limited [15,16].

Children’s patient education need to be based on development psychology to support their age-appropriate use of knowledge [6]. As for 6-12-year-old school-age children, they are motivated to learn, they have physical coordination and dexterity, their ability to think logically develops, but individual differences in development occur [17,18]. In teaching, concrete terminology and step-by-step instructions should be used, and play or other demonstration materials prove useful [17]. Children need to have opportunities to practice skills in a safe atmosphere and be rewarded for their accomplishments [18]. Although school-age children learn technical glucose monitoring skills, they need their parents to provide emotional support, physical assistance, and management guidance [5,17]. Therefore, parent participation proved essential in school-age children’s diabetes education [5,6].

Although no preferred diabetes education approach exists [19], it is important to re-establish a sense of control and school-age children [20,21] and parents [22-24] benefit from being involved in the care and patient education.

In general, diabetes education is based on structured curricula comprising survival skills upon diagnosis and continuing education later on [6,25]. The survival skills include basic information on diabetes, insulin therapy, blood glucose levels and targets, physical exercise, and hypoglycemia. It also incorporates practical skills such as blood glucose monitoring, insulin dose calculation, administrating and injecting, as well as diet management [2,6,25]. In Finland, the survival skills are usually taught as in-patient education of both parents and the child. To learn by doing gives the child and parents a growing sense of confidence and competence as well as supports the emotional coping with the diagnosis at the same time [6,24].

## Background

Each topic of the diabetes curricula is taught in an educational session and thereafter it is trained in practice under the guidance of nurses. In this educational program, we focused on a module of the survival skills, the blood glucose monitoring education and the empowering way to provide it. The purpose of this article was to describe an educational program to enhance empowering patient education process for the blood glucose monitoring education of school-age children and nurses’ perceptions of using empowering techniques.

## Methods

### Study tool

**Figure 1:**
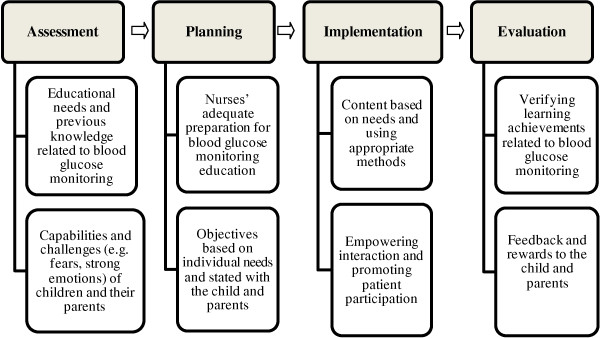
Content of the manual for empowering patient education.

This educational program is part of a project aiming to develop patient education in pediatric wards. The project team composed of research (N = 2), patient education (N = 2), pediatric nursing (N = 5), diabetes management (N = 2) experts, and the parents of children with a chronic illness (N = 2) representing patient associations. The project team assembled for monthly meetings to review the learning theories and empowering patient education theories that aided in the development. The educational program was based on the principles of teaching school-age children and on the patient education process conducted in an empowering way. The program was developed based on literature and prior research as well as two studies focusing on nurses’ [12] and school-age children’s and parents’ [15] perceptions of patient education. In addition, information from the local context was acquired by consulting the pediatric diabetes nurse specialists and the pediatrician of the hospital unit. The blood glucose monitoring education is illustrated in Figure 1.

The empowering patient education process for the blood glucose monitoring education of school-age children was carried out in a pediatric ward of a Finnish hospital. The ward provided care for children with diabetes or other pediatric diseases. In education workshops in February 2010, the educational program was presented to the ward nurses and the pediatric diabetes team of the hospital. During one day, the pediatric nurse teachers of the project team taught nurses to carry out the blood glucose monitoring education using an empowering method of teaching school-age children and their parents. Teaching was provided with interactive methods and by using practical case-solving. All nurses also received the educational material including a manual explaining how to proceed in each phase of the patient education process.

After the nurses’ education, the first author worked in close cooperation with the nurses and motivated them to use empowering techniques when a school-age child received the diabetes diagnosis, and the blood glucose monitoring education was given to the child and parents. In addition, the key issues of the patient education program were repeated to the nurses in monthly workshops.

### Ethics and study population

The management of the hospital granted the study permission, and its research ethics committee approved the study. Altogether 10 nurses carried out the blood glucose monitoring education according to the program. Three of them did it twice and were interviewed twice. However, in two cases the interview could not be conducted, and thus the data comprised a total of 11 descriptions of the blood glucose monitoring education implemented by 8 nurses. All nurses gave a written consent for their participation. The nurses’ age varied from 25 to 55, and their working experience in the ward ranged between 6 months and 13 years.

### Study of Nurses’ perceptions

During the year following the nurses’ education, their perceptions of utilizing the empowering patient education were explored to further develop the educational program. The study aimed to describe (1) the nurses’ perceptions of successful empowering blood glucose monitoring education of school-age children, and (2) the challenges nurses faced in it.

The first author collected the data by semi-structured interviews. Their themes were based on the various phases of the patient education process and interaction. After the nurse had taught blood glucose monitoring to a school-age child, the nurse was interviewed in a tranquil setting. The tape-recorded interviews lasted from 17 to 36 minutes and were transcribed verbatim by the interviewer.

First, the data were analyzed by a deductive content analysis [26,27]. The patient education process formed the structure of the analysis. The expressions describing empowering education techniques were coded into 4 main categories indicating the phases of the patient education process. Each main category was then reviewed, and the content was divided into 9 subcategories. The subcategories were created following the principles of inductive content analysis and were named to illustrate their content. Second, the challenges of managing empowering patient education were analyzed inductively [26,27]. All expressions of challenges were identified, and the expressions with similar characteristics were grouped in subcategories forming the main categories. By the end of the analysis, 3 main categories emerged. The first author analyzed the data, during which time she conferred intensively with the research group reviewing the analysis process. Since the analysis of the successful management of empowering education and the challenges was conducted independent from each other, one description in the data could be utilized for depicting both empowering techniques and challenges. The results are presented in Table 1 and 2 showing the subcategories and the number of the descriptions.

**Table 1:** Nurses’ descriptions of empowering patient education process for blood glucose monitoring

**Main category**	**Subcategory (N = number of descriptions)**	**Content of the subcategory**
Need assessment	Multiple methods (N = 8)	Observation of the child and parents and their capabilities
		Asking questions and interviewing the family
		Reviewing hospital documents or consulting the primary nurse
	Content (N = 7)	Previous knowledge and skills
		Previous experience of hospitalization; fears, shock or other mental distress
		Personal character and family interaction or conflicts
		Everyday life (hobbies, day care and parental involvement)
Planning	Adequate preparation (N = 8)	Relevant information on the patient, since education provided by the primary nurse
		Preserving privacy in patient education and tranquil setting
		Appropriate individual material and equipment
		Considering methods in advance for school-age child and parents
	Multiple objectives discussed with the child and parents (N = 7)	Cognitive objectives (e.g. knowing target levels; understanding reasons for testing)
		Capability objectives (e.g. managing techniques for blood glucose testing)
		Experiential objectives (e.g. measuring not a scary experience for the child)
		Attitude objectives (e.g. family’s readiness for practicing measuring)
Implementation	Content of teaching (N = 10)	Techniques for blood glucose testing
		Information on blood glucose levels, measurement times and sources of errors in measuring
		Information on what values mean and what to do with them
		Keeping record of home monitoring
	Use of teaching materials (N = 6)	Written material delivered when hospitalized
		Pictures, equipment and other demonstrative materials selected for child and parents
		Individual material for keeping record of home monitoring
	Teaching methods (N = 7)	Interactive education (e.g. arranging friendly atmosphere; showing approval and empathy; verbal counseling combined with demonstration by play, pictures and equipment; instructing step by step; encouraging child and parents’ practical training; repeating instructions when needed)
		Child-centered approach (e.g. encouraging child to participate by overcoming fear; respecting child’s own will by offering options to participate; teaching age-appropriately)
		Family-centered approach (e.g. encouraging parents to ask questions, listening to them and answering questions; teaching child and parents together; proceeding on family’s terms; giving feedback)
Evaluation	Various methods (N = 10)	Nurse’s self-evaluation
		Impression of the situation and observation of child and parents’ actions
		Asking questions and discussion on feelings
		Asking child and parents to explain what they learned and express their capability for monitoring
		Using knowledge test or other evaluation measurements
	Documentation (N = 10)	Patient education form and patient files
		Subject of patient education and participants
		Reactions of child and parents
		Level of learning and need for repetition

**Table 2:** Challenges nurses faced in managing empowering patient education

**Condensed meaning**	**Subcategory (N = number of descriptions)**	**Main category**
Child/family not familiar to the nurse	Lack of information and expertise (N = 7)	Management and leadership challenges
Only limited experience of diabetes education in the diagnostic phase		
Not enough information on the empowering patient education process		
Being not sure of one’s ability to include all the details of empowering patient education		
Wondering if educating differently from colleagues		
Interruptions during education caused by colleagues seeking advice	Lack of vital resources (N = 4)	
Education took place in a room with other families and without patient privacy		
Lack of appropriate demonstrative material for school-age children		
Teaching the traditional blood monitoring education	Nurse-centered education in practice (N = 6)	Ambivalence with traditional and empowering patient education
Not discussing objectives with the family, but knowing they should be communicated		
Tendency to teach all parents in the same way		
Using partly non-interactive teaching in which only the child tested blood glucose		
Tendency to dominate the discussion		
Education should be based more on the child and parents’ needs	Identification of the need for child- and family-centered education (N = 6)	
Child and parent participation should be increased by listening to them and negotiating with them		
Child not taking the disease seriously	Child as passive participant (N = 2)	Challenges of child or parent’s situation
Child is quiet and refusing to measure blood glucose		
Parent not being present enough and unwilling to listen to anything	Parents suffering from shock (N = 4)	
Parents remain passive and not able to receive or utilize any information		
It is difficult to judge from parents’ behavior if they understood anything		

## Results

### Successful managing of the empowering patient education process

The successful managing of the empowering patient education process consisted of 4 categories: need assessment, planning, implementation, and evaluation (see Table 1). The need assessment employed various methods for gathering information, such as observation, asking questions, and reviewing hospital documents. The content of this information, including the participants’ previous knowledge, skills and experiences, was considered in the education. Planning comprised 2 elements: First, adequate preparation aimed at individualizing the education by providing a private room and age-appropriate materials as well as considering the content and methods in advance. Second, multiple objectives were established with the family. The objectives included cognitive, capability, experiential and attitude objectives related to blood glucose monitoring.

The nurses’ descriptions of the implementation comprised information on the content, teaching materials and methods. The content of patient education was based on the objectives and contained information related to blood glucose control and techniques for blood glucose testing. Teaching material was to meet the individual needs of the children and their parents. Different materials were used, such as equipment, demonstrative and written material. Patient education was implemented using different teaching methods: First, interactive education included creating friendly atmosphere, showing approval, encouraging the child and parents, and using verbal counseling combined with other methods. Second, child-centered approach focused on the patient and promoted his/her participation by respecting the child, utilizing the child’s natural curiosity, helping to overcome one’s fear, and using simple language as well as rewards. Third, family-centered approach supported parent participation by listening to them and answering questions, teaching all family members together, proceeding on their terms, and giving feedback.

The evaluation consisted of various methods and documentation. The methods, such as observing, asking questions, asking to explain the learning, and discussing feelings, promoted patient participation. The documentation of patient education including the subject of the session, participants, level of learning and need for repetition, was completed in a patient education form and patient files.

### Challenges of managing empowering patient education

The challenges formed 3 categories (Table 2). First, the management and leadership challenges comprised lack of information and expertise, including uncertainty about implementing the empowering patient education process and lack of vital resources, such as time, place and material. Second, ambivalence with traditional and empowering patient education was shown. In practice, the nurse-centered approach to patient education manifested itself in nurses knowing the principles of empowering patient education, but not always applying them to teaching. In addition, nurses identified the need for child- and family-centered patient education which focused on the child’s and parents’ needs and was implemented in cooperation with them. Third, the challenges facing the child’s or parents’ overall situation were recognized. In some cases, the child remained a passive participant in the patient education, and the parents were in a state of shock limiting their ability to participate in it.

## Discussion

In this paper, we described an educational program aimed to enhance nurses’ competence in empowering diabetes education. We also reported the nurses’ perceptions of the empowering education process. In successful empowering patient education, the need assessment was conducted comprehensively by using multiple methods for collecting information. Further, planning was done adequately for individualized patient education and the objectives including cognitive, capability, experiential and attitude objectives were stated with the family. This is opposed to previous studies [10,11] revealing deficiency in the need assessment and planning.

The principles of empowering patient education [4,12,14] as well as child and parent participation [6,17,20-24] emerged in the implementation phase of the patient education process. In this study, the content of patient education was based on individual needs and objectives to enable the child and parents to master the knowledge and skills of blood glucose monitoring. Similarly, the teaching material was selected to meet the needs of the child and parents. The teaching methods, including interactive education, and child- and family-centered approach promoted patient participation.

The patient education was evaluated appropriately by verifying the learning achievements with multiple methods, and the documentation of patient education was produced accurately. This is in contrast to earlier research [10,11] observing limited evaluation strategies and documentation. In this pilot, the nurses seemed to adopt the empowering approach described in the manual to carry out patient education.

Three types of challenges were met in managing empowering patient education. First, challenges were related to management and leadership: lack of information and expertise as well as lack of vital resources. These issues indicate that teaching as such is not enough when targeting changes in work practices. Contextual factors related to a social system as well as organisations’ management and leadership issues play a key role in development [28]. The management’s responsibility for patient education is to provide continuity of education, adequate settings for private education, and effective information channels.

Second, the ambivalence with traditional and empowering patient education revealed that the empowering patient education approach was adopted intellectually. However, changing the old intrinsic patient education paradigm was difficult despite the need for change being recognized. This change does not occur at once but it takes time. A positive and safe atmosphere for development as well as reflective team activities can promote changes in work practices [28].

Third, challenges of client’s situation emerged when the child was a passive participant, or the parents suffered from a shock. Factors impacting teaching included the stress of a chronic illness and anxiety as well as the negative influence of the hospital environment, resulting in loss of control [13]. In these situations, expressions of emotional support, such as being available, maintaining hope and giving only convincing and matter-of-fact information, could prove beneficial. Children and parents can benefit from individualized care and flexible schedule for patient education as well as support from a mental health professional.

The interviewed nurses described successful management of the empowering patient education suggesting that this study produced a useful evidence-based patient education program for diabetes education. On one hand, the nurses’ interviews indicate that a checklist of the content of the empowering patient education process could help nurses to prepare for and focus on the critical issues in the blood glucose monitoring education. On the other hand, the challenges nurses faced in managing empowering patient education showed that teaching nurses as such is not enough when aiming at changes in nursing practices. Therefore, the challenges revealed in this study should be considered when new educational programs are planned and implemented.

### Study limitations

Our study has some limitations. The number of school-age children with a recent diabetes diagnosis remained smaller than was assumed, and the nurses had only a few possibilities to implement empowering patient education. Moreover, the nurses’ interview data were the only source of information. The data revealed nurses’ descriptions of empowering patient education, but additional data and methodological triangulation could have given a more comprehensive description of what really happened during the patient education process.

### Implications for clinical practice and further research

This paper provides an example of developing an evidence-based patient education program that can be useful for those planning and carrying out improvements in nursing practices and for managers and leaders in nursing units. The study provides practical examples of empowering patient education beneficial for all health care professionals working with school-age children and their parents. The nurses’ manual can add a valuable asset when new colleagues and nursing students are instructed to use the methods and content of empowering diabetes education. In addition, the findings may be utilized in pediatric units to develop materials and distribute resources as well as increase nurses’ capabilities. The study describes the challenges that need to be considered when aiming to change work practices. Teaching nurses should be combined with other strategies for discerning possible barriers for implementing the educational program.

A rather small number of nurses implemented the educational program for school-age children with diabetes, and research is needed to obtain a more credible picture of the program and to develop it further. We also need more research to understand the usefulness of the program. Thus, observing the patient education sessions would extend our knowledge of them. The evaluation of a patient education program should involve the children’s and their parents’ perspective, and it would be useful to collect information about their learning achievements.

## Competing interests

The authors declare that they have no competing interests.

## Authors’ contributions

MK was responsible for the study design, carried out data collection and analysis and drafted the manuscript. EE and IE participated in the design of the study, reviewed the data analysis, were involved in the drafting of the manuscript and contributed to the critical revision of important intellectual content of the manuscript. All authors read and approved the final manuscript.

## References

[B1] American Diabetes Association Standards of medical care in diabetes–2011 Diabetes Care 2011 34 Suppl 1 s11 s61 21193625 10.2337/dc11-S011 PMC3006050

[B2] Silverstein J Klingensmith G Copeland K Care for children and adolescents with type 1 diabetes. A statement of the American Diabetes Association Diabetes Care 2005 28 186 212 10.2337/diacare.28.1.186 15616254

[B3] Funnell MM Anderson RM Arnold MS Empowerment: An idea whose time has come in diabetes education Diabetes Educ 1991 17 37 41 10.1177/014572179101700108 1986902

[B4] Anderson RM Funnell MM Patient empowerment: Myths and misconceptions Patient Educ Couns 2010 79 277 282 10.1016/j.pec.2009.07.025 19682830 PMC2879465

[B5] Kelo M Martikainen M Eriksson E Self-care of school-age children with diabetes: An integrative review J Adv Nurs 2011 67 2096 2108 10.1111/j.1365-2648.2011.05682.x 21635284

[B6] Lange K Sassmann H von Schütz W Kordonouri O Danne T Prerequisites for age-appropriate education in type 1 diabetes: a model programme for paediaric diabetes education in Germany Pediatr Diabetes 2007 8 Suppl 6 63 71 17727387 10.1111/j.1399-5448.2007.00277.x

[B7] Llahana SV Poulton BC Coates VE The paediatric diabetes specialist nurse and diabetes education in childhood J Adv Nurs 2001 33 296 306 10.1046/j.1365-2648.2001.01665.x 11251716

[B8] Peimani M Tabatabaei-Malazy O Pajouhi M Nurses’ role in diabetes care; A review Iranian Journal of Diabetes and Lipid Disorders 2010 9 1 9

[B9] Carpenter JA Bell SK What do nurses know about teaching patients? J Nurses Staff Dev 2002 18 157 161 10.1097/00124645-200205000-00009 12189998

[B10] Barber-Parker ED Integrating patient teaching into bedside patient care: a participant-observation study of hospital nurses Patient Educ Couns 2002 48 107 113 10.1016/S0738-3991(02)00024-1 12401413

[B11] Turner dS Wellard S Bethune E Registered nurses’ perceptions of teaching: Constraints to the teaching moment Int J Nurs Pract 1999 5 14 20 10.1046/j.1440-172x.1999.00147.x 10455612

[B12] Kelo M Martikainen M Eriksson E Patient education of children and their families: nurses’ experiences Pediatr Nurs 2013 39 71 79 23705298

[B13] Bastable SB Bastable SB Overview of education in health care Nurse as educator: Principles of teaching and learning practice 2008 3 Sudbury, MA: Jones and Bartlett Publishers pp. 3 23

[B14] Aujoulat I d’Hoore W Deccache A Patient empowerment in theory and practice: Polysemy or cacophony? Patient Educ Couns 2007 66 13 20 10.1016/j.pec.2006.09.008 17084059

[B15] Kelo M Eriksson E Eriksson I School-age children’s and their parents’ perception of nurses as educators Scand J Caring Sci 2012 10.1111/scs.12001 23088248

[B16] Pyörälä E The participation roles of children and adolescents in the dietary counseling of diabetics Patient Educ Couns 2004 55 385 395 10.1016/j.pec.2003.04.008 15582345

[B17] Bastable SB Dart MA Bastable SB Developmental stages of the learner Nurse as educator: Principles of teaching and learning practice 2008 3 Sudbury, MA: Jones and Bartlett Publishers pp. 147 198

[B18] Santrock JW Child development 2007 11 Boston: McGraw-Hill.JW

[B19] Funnell MM Brown TL Childs BP Haas LB Hosey GM Jensen B National standards for diabetes self-management education Diabetes Care 2008 31 Suppl 1 97 103 10.2337/dc08-S097 PMC2797381 18165344

[B20] Herrman JW Children’s and young adolescents’ voices: perceptions of the costs and rewards of diabetes and its treatment J Pediatr Nurs 2006 21 211 221 10.1016/j.pedn.2005.07.012 16713511

[B21] Pelander T Leino-Kilpi H Katajisto J Quality of pediatric nursing care in Finland. Children’s perspective J Nurs Care Qual 2007 22 185 94 10.1097/01.NCQ.0000263110.38591.9a 17353757

[B22] Hallström I Elander G Families’ needs when a child is long-term ill: a literature review with reference to nursing research Int J Nurs Pract 2007 13 193 200 10.1111/j.1440-172X.2007.00625.x 17518793

[B23] Hummelinck A Pollock K Parents’ information needs about the treatment of their chronically ill child: A qualitative study Patient Educ Couns 2005 62 228 34 16139981 10.1016/j.pec.2005.07.006

[B24] Wennick A Hallström I Swedish families’ lived experience when a child is first diagnosed as having insulin-dependent diabetes mellitus: an ongoing learning process J Fam Nurs 2006 12 368 389 10.1177/1074840706296724 17099116

[B25] Swift PGF Diabetes education in children and adolescents Pediatr Diabetes 2009 10 Suppl 12 51 7 19754618 10.1111/j.1399-5448.2009.00570.x

[B26] Elo S Kyngäs H The qualitative content analysis process J Adv Nurs 2008 62 107 115 10.1111/j.1365-2648.2007.04569.x 18352969

[B27] Patton MQ Qualitative research and evaluation methods 2002 3 Thousand Oaks, California: Sage Publications

[B28] Titler M Translating research into practice. Models for changing clinician behavior Am J Nurs 2007 107 Suppl 26 31 17563431 10.1097/01.NAJ.0000277823.51806.10

